# Evidence of spatial periodic firing in the subiculum of mice

**DOI:** 10.3389/fncir.2025.1648844

**Published:** 2025-11-25

**Authors:** P. Abad-Perez, G. Rigamonti, F. J. Molina-Paya, G. Cabral-Pereira, Miriam Esteve-Campello, R. Scott, V. Borrell, L. Martínez-Otero, A. Falco, J. R. Brotons-Mas

**Affiliations:** 1 Universidad Cardenal Herrera-CEU, CEU Universities, Valencia, Spain; 2 Instituto de Neurociencias UMH-CSIC Alicante, Sant Joan d'Alacant, Spain; 3 Grupo de Investigación Multidisciplinar en la enfermedad de Alzheimer UMH-UCHCEU, Alicante, Spain; 4 Universidad de Alicante, Alicante, Spain

**Keywords:** subiculum, spatial coding, grid-like firing, place cells, boundary-vector cells, corner cells, bursting, proximodistal gradient

## Abstract

The subiculum is a critical node of the hippocampal formation, integrating multiple circuits—including thalamic inputs and afferents from CA1 and medial entorhinal cortex—and projecting broadly to cortical and subcortical targets. Yet its contribution to spatial coding remains incompletely understood. We recorded single-unit activity in freely moving mice using two complementary electrophysiological approaches: (i) chronic tetrodes targeting CA1 and the dorsal subiculum (SUB), and (ii) 64-channel linear silicon probes targeting dorsal SUB. In addition to place cells, boundary-vector cells (BVCs) and corner cells (CCs), we identified a subset of subicular neurons that exhibited spatially periodic, grid-like firing patterns. This phenomenon was replicated across recording technologies, indicating that periodic coding is a consistent feature of the mouse subiculum. Compared with CA1 place cells, SUB spatial neurons exhibited lower spatial information and reduced within-session stability, suggesting distinct coding regimes across hippocampal subregions. Sampling along the proximodistal axis with probe arrays further revealed that burst propensity correlated positively with spatial information at more distal recording sites, consistent with known physiological gradients in subiculum and echoing relationships seen in CA1. Together, these results expand the repertoire of identified spatial codes in SUB and support the view in which subiculum contributes to geometry- and periodicity-based representations that complement CA1 and entorhinal spatial coding, thereby shaping downstream computations in cortico-subcortical circuits.

## Introduction

Since the discovery of hippocampal place cells—neurons that code for space by being active in specific locations of the environment (O’Keefe and Dostrovsky, 1971)—numerous studies have aimed to understand the computational mechanisms of spatial navigation. As a result, different types of spatially modulated neurons have been identified across brain regions: head direction (HD) cells; border and boundary-vector cells (BVCs), which encode environmental geometry in the medial entorhinal cortex (MEC) and the SUB; and grid cells (GCs) in the MEC (Hafting et al., 2005; Fyhn et al., 2008; Taube et al., 1990; Lever et al., 2009; Solstad et al., 2008; Sharp and Green, 1994; Brotons-Mas et al., 2017). These neurons are strongly involved either in the codification of external information—such as BVC coding for geometry, or in encoding egocentric-related information. This is the case for grid cells (GCs), where tessellating regular firing is associated with the representation of spatial metrics and is, therefore, essential for path integration—the ability to navigate in the absence of external cues (McNaughton et al., 2006). The integration of both allocentric and egocentric streams of information is instrumental in generating an efficient spatial representation.

Numerous computational models have aimed to uncover the mechanisms underlying the integration of different types of spatial signals. The BVC model suggested that place-cell activity depends on the existence of neurons coding for the geometry of the environment (Hartley et al., 2000). Other models suggested that place cells require GC activity (Hafting et al., 2005; O’Keefe and Burgess, 2005). However, single-unit recordings during neurodevelopment demonstrated that HD, place cells, and boundary neurons appear before GC during neurodevelopment (Wills et al., 2010; Langston et al., 2010). Thus, the dependence of place-cell activity on grid cell firing seems to be unclear (Morris and Derdikman, 2023).

Further research has examined the heterogeneity of spatial cell types and their distributed anatomical locations. In this context, the SUB may serve as a critical anatomical hub for spatial navigation (Aggleton and Christiansen, 2015; O’Mara et al., 2009). Strategically located between the entorhinal cortex (EC) and CA1, the SUB supports spatial information processing in a complex and heterogeneous manner. While spatial neurons in CA1 typically exhibit spatial reliability, subicular neurons encode multiple behavioral correlates—including location, speed, direction, and the geometry of the spatial context—while providing reliable spatial representation across light–dark transitions (Sharp, 1997; Brotons-Mas et al., 2010; Barnes et al., 1990; Ledergerber et al., 2021). The SUB contains neurons with diverse electrophysiological characteristics that might have different roles in memory processing (Eller et al., 2015; Jarsky et al., 2008).

Previous work in rats indicated the existence of grid-like, spatially tuned activity in the SUB, attributed to axonal inputs from the MEC (Brotons-Mas et al., 2017). This raised the possibility that subicular pyramidal neurons could integrate periodic signals from the EC and aperiodic signals from CA1. However, whether such grid-like activity originates from local subicular pyramidal neurons has not been extensively investigated until now. Here, we sought to test the presence of periodic firing in individual subicular cells of mice using large open-field arenas. We found evidence of grid-like and other forms of spatially periodic firing (i.e., band-like; Krupic et al., 2012) in subicular neurons whose electrophysiological characteristics are compatible with those of pyramidal neurons. To the best of our knowledge, this is the first evidence of spatially periodic neurons in the mice SUB, highlighting the potential role of this structure in shaping the spatial cognitive map.

## Methods

### Subjects

Male wild-type C57BL/6 mice (*N* = 11; aged p60–p90) obtained from the UMH “Servicio de Experimentación Animal (SEA)” were used in this study. Mice were maintained on a 12-h light/dark cycle with food and water available *ad libitum* and were individually housed following electrode implantation. All the procedures were approved by the UMH-CSIC Ethics Committee and the regional government and complied with local and European guidelines for animal experimentation (86/609/EEC).

### *In vivo* recordings on freely moving mice

**Figure 1:**
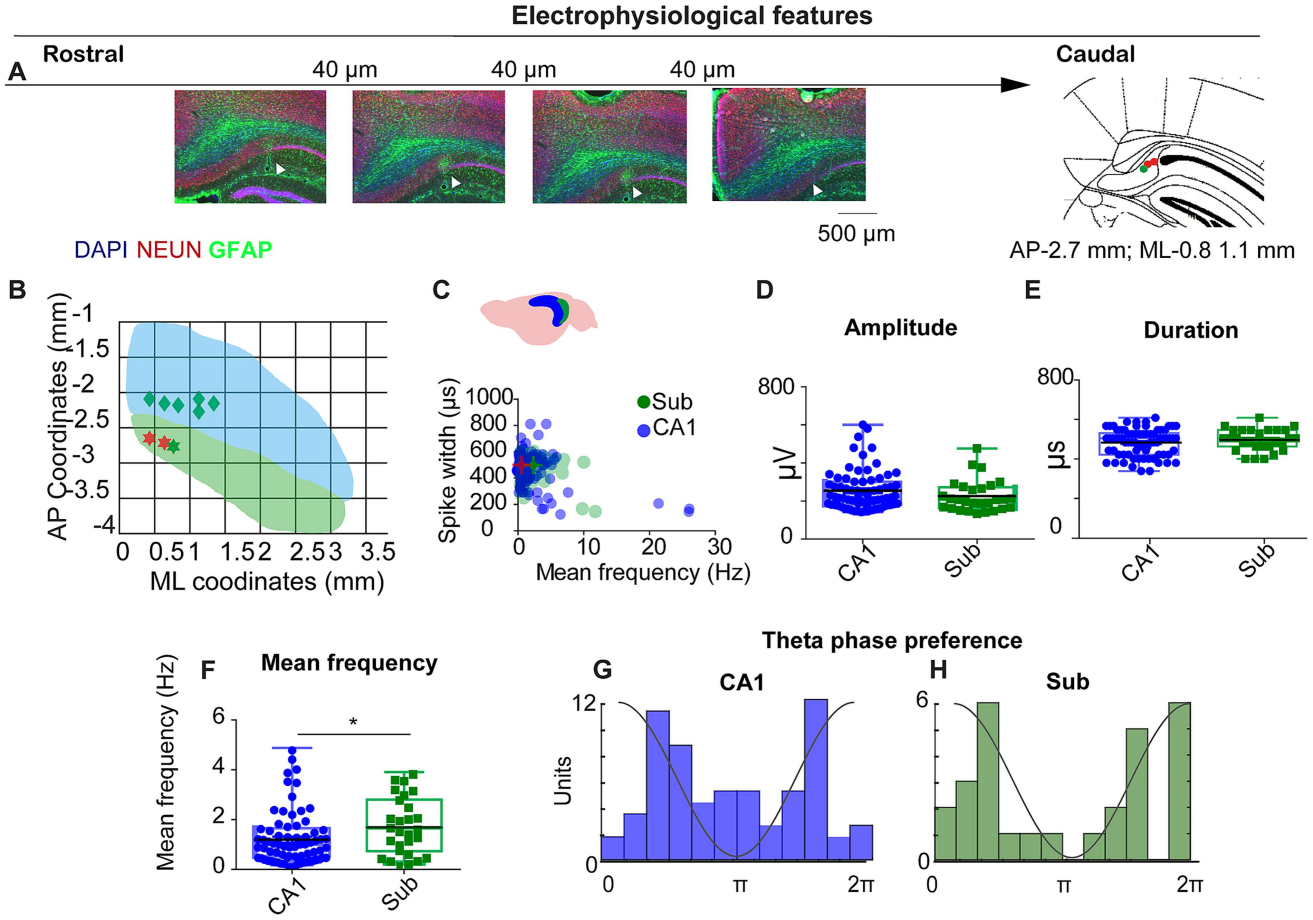
Electrophysiological features of CA1 and subiculum recordings. **(A)** Histological verification of tetrode tracks across the rostro-caudal extent of dorsal subiculum (DAPI/NEUN/GFAP). Arrowheads, electrode tip; schematic at right shows example target (AP − 2.7 mm; ML + 0.8–1.1 mm). Scale bars as indicated. **(B)** Recording coverage in mediolateral (ML) and anteroposterior (AP) coordinates; green = SUB, blue = CA1. **(C)** Scatter of spike width (trough-to-peak, μs) vs. mean firing rate (Hz) for CA1 (blue) and SUB (green). **(D–F)** Group comparisons for spike amplitude **(D)**, spike duration **(E)**, and mean firing rate **(F)**. SUB neurons fired faster than CA1 (see Results). **(G,H)** Theta-phase preference histograms (4–12 Hz) for CA1 **(G)** and SUB **(H)**; CA1 shows bimodality with a concentration near trough; SUB preferentially fires near the theta peak.

Custom-designed microdrives (Axona Ltd.) carrying twisted-wire tetrodes (12-μm tungsten, California Fine Wire, Grover Beach, CA, United States) were used to target the dorsal CA1 and dorsal subiculum. Under 1.5% isoflurane anesthesia and peri-operative buprenorphine (0.05 mg/kg, s.c.), small craniotomies were performed over the dorsal hippocampus and dorsal subiculum. Guide cannulas were aimed at dorsal CA1 (AP − 2.2 mm, ML + 1.0 mm from bregma) and predominantly proximal dorsal subiculum (AP − 2.7 mm, ML + 0.5 mm; right hemisphere; Figures 1A,B). Of the nimals implanted with microdrives, three met the anatomical/physiological criteria for inclusion in both CA1 and SUB datasets. Because tetrodes advance along a single guide, coverage along the proximodistal axis was limited. To increase both the number of recorded neurons and proximodistal coverage, a separate cohort of two mice was implanted with 64-channel linear silicon probes (NeuroNexus, Ann Arbor, MI, United States) targeting the dorsal subiculum (AP − 3.8 mm, ML + 1.5 mm), with coordinates chosen to maximize subicular coverage. The linear arrays (≈800 μm span) enabled sampling from proximal to more distal positions within dorsal subiculum. Electrode depths (tetrodes and probes) were adjusted across days until characteristic hippocampal/subicular activity was observed.

### Data acquisition

Tetrode recordings. As in our previous study (Del Pino et al., 2017), recordings were obtained using a 16-channel headstage (gain x1), with an infrared light-emitting diode (LED) for tracking mouse position (Axona Ltd., United Kingdom). Signals were amplified (400 to 1,000x), band-pass filtered (0.3 Hz to 24 kHz; Dacq system, Axona, United Kingdom), and recorded at 48 kHz with 24-bit precision. For single-unit recordings, the signal was band-pass filtered between 380 Hz and 6 kHz. Spikes were recorded whenever the signal was 3–4 times above background noise, typically 20–30 μV, and stored in windows of 1-ms duration (200 ms before threshold and 800 ms after threshold detection). Spike waveforms were sampled at 48 kHz, while the same channels were also recorded in continuous mode at 4.8 kHz and band-pass filtered between 0–3 and 2.4 kHz.

Silicon-probe recordings. In line with our previous study (Valero et al., 2025), high-density recordings were obtained using an Intan-based system (NeuroNexus, Ann Arbor, MI, United States) at 30 kHz with 16-bit resolution. The wideband stream was used for spike sorting, while a downsampled copy at 1.25 kHz served as the local field potential (LFP) signal. During recording sessions, electrode advancement and target verification relied on electrophysiological landmarks—including ripple power, unit activity, and theta power—to optimize depth and confirm subicular/hippocampal placement.

### Place cells recordings

Mice performed a pellet-chasing task. Animals were maintained under mild food restriction, kept at ≤20% below their free-feeding weight. During recording, small food pellets were tossed pseudo-randomly into the arena at ~20-s intervals to sustain locomotion and promote uniform spatial sampling. All mice were habituated to each arena for several days before recording. Tetrode-implanted mice were recorded in a 50 × 50 cm arena (20 min) and in a larger 70 × 70 cm arena (30 min). The silicon-probe cohort was recorded in an 80 × 80 cm arena (30–40 min) to maximize the chances of detecting grid/periodic firing.

### Data analysis

LFP data were analyzed using custom-written MATLAB codes (Mathworks, Natick, MA, United States). Raw recordings were FIR-filtered (<1.2 kHz) and downsampled to 2.4 kHz. Similar to previous studies (Del Pino et al., 2017; Del Pino et al., 2013), data obtained from open-field recordings were used to characterize the LFP. To visualize the relationship between spectral power and movement speed, the spectral power (in decibels, 10log_10_) and spectrogram were computed using the Thomson multi-taper method. A sliding window with 50% overlap, yielding a frequency resolution of 1 Hz, was used.

### Unit isolation and spike train analysis

Single-unit activity from tetrode recordings was isolated with TINT (Axona, St. Albans, United Kingdom). This software is specifically designed for tetrode data cluster sorting. Feature vectors included the first principal components, peak-to-trough duration, peak-and-trough amplitudes, time of peak and trough, and waveform energy on each wire. KlustaKwik was used for initial automated clustering, followed by manual curation in TINT based on principal components, spike amplitude, and waveform consistency. Cluster quality was quantified by the overlap probability metric as described previously (Del Pino et al., 2017; Hill et al., 2011). Spike trains were analyzed by generating interval time histograms and temporal auto-correlogram. Only units with no spikes in the refractory period of the inter-spike time histogram (1–2 ms), and with spike amplitudes 3–4 times above background noise, typically 20–30 mV, were included. Putative pyramidal cells and interneurons were distinguished based on waveform duration and firing rate characteristics, as previously described (Brotons-Mas et al., 2017). Further spike train analyses were conducted to determine the bursting properties of the recorded neurons, using the burst index (BI) proposed by Royer and the propensity to burst (PrtB) proposed by Staff (Royer et al., 2010; Staff et al., 2000).

For high-density recordings with silicone probes, we followed the same approach as in our previous work (Valero et al., 2025). In brief, spike sorting was performed semi-automatically with KiloSort^1^ via the KilosortWrapper pipeline,^2^ as in (McNaughton et al., 2006; Hartley et al., 2000; Long et al., 2014). Resulting clusters were manually refined in Phy^3^ using community plug-ins.^4^ The following parameters were used for KiloSort: pos. Nfilt: 6 *numberChannels; ops.nt0: 64; ops.whitening: ‘fulll’; ops.nSkipCov: 1; ops.whiteningRange: 64; ops.criterionNoiseChannels: 0.00001; ops. Nrank: 3; ops.nfullpasses: 6; ops.maxFR: 20000; ops.fshigh:300; ops.ntbuff: 64; ops.scaleproc: 200; ops. Th: [4 10 10]; ops.lam: [5 20 20]; ops.nannealpasses: 4; ops.momentum: 1./[20,800]; ops.shuffleclusters: 1. Electrophysiological neuron classification was performed using CellExplorer^5^ (Petersen et al., 2021). This algorithm classifies units using two main features: spike waveform width (trough-to-peak, TTP) and burstiness derived from the autocorrelogram (ACG). Using the waveform and burstiness criteria, units are tentatively segregated into narrow waveform (trough-to-peak ≤ 450 μs) and wide waveform (trough-to-peak > 450 μs and τ_rise_ > 6 ms) putative interneurons, putative interneurons, and the rest, pyramidal neurons or unclassified. Because tetrode spikes were extracted from thresholded high-pass snippets, whereas probe spikes were extracted from broadband continuous data, we did not compare absolute waveform amplitudes across technologies. All downstream spatial metrics—rate-map construction, information, coherence, and stability—as well as grid/periodicity analyses were identical for tetrode and probe datasets.

### Phase locking to ongoing oscillations

Phase locking of single units to ongoing oscillations was computed as previously described (Valero et al., 2025; Valero et al., 2022). The analysis toolbox is available online.^6^ The LFP was filtered for each frequency of interest, and the Hilbert transform was applied to obtain the instantaneous phase. Phase locking was computed by calculating the phase angles for each timestamp where an action potential occurred. A histogram of phase angles was calculated, and the circular mean and resultant vector were calculated for each neuron (Berens, 2009). Only neurons that were significantly modulated in each frequency band during the baseline condition (Rayleigh test) were included in the analysis.

### Firing rate maps

In the case of the tetrode recordings, firing-rate maps were assembled as described previously (Brotons-Mas et al., 2017; Pérez et al., 2023). Pixel maps were converted to a bin matrix with a bin size of 2.5 cm x 2.5 cm. Firing rate in each bin was determined by a smoothing process using overlapping squares of 7.5 cm x 7.5 cm, as described before. Firing fields were plotted as a contour map reflecting the frequency of firing; colors were interpolated from the highest to the lowest firing bins by scaling in decreasing intervals of peak firing, with a color scale ranging from red for the highest firing rates to dark blue for the lowest. A similar approach was implemented with multisite experiments; however, in this case, the FMA toolbox was used.^7^

For both sets of data, the spatial information content per spike and per second was quantified using the Skaggs information indexes for the smoothed map (Markus et al., 1994). In addition, spatial coherence, a spatial autocorrelation measurement, was calculated as the correlation coefficient between the firing rate of each bin and the average rate of its eight surrounding bins (Muller and Kubie, 1987). Firing fields were defined as a group of six contiguous bins in which the firing frequency exceeded the mean firing frequency plus the standard error of the firing matrix. The maximal firing frequency of this group of bins had to be above 1 Hz. For those units displaying more than one firing field, firing field size was computed as the sum of the existing firing fields and expressed as the percentage of the arena size occupied by the firing field, calculated using the smoothed firing matrix. The in-field maximum frequency was defined as the highest firing rate within a firing field on the smoothed firing map. Mean frequency was calculated by dividing the total number of spikes by the total recording time, yielding the average session firing rate in Hz.

To classify units as having spatially related activity, a randomized distribution was generated by circularly shifting the spike times relative to the position. This strategy allowed us to generate a random distribution without affecting the temporal firing structure of neurons (Langston et al., 2010). The values were obtained from the original, unsmoothed firing map while preserving the original trajectory of mice (*n* = 1,000). For a unit to be regarded as being spatially modulated, it had to display a spatial coherence above 95% of the randomized distribution. This same random distribution was used to obtain the intratrial stability index between the original and a random distribution. Thus, units were considered stable if their cross-correlation between the original and the randomized distributions exceeded the 95^th^ percentile.

### Spatial typology analysis

Multiple spatial response types have been reported in subiculum (Lever et al., 2009; Brotons-Mas et al., 2017; Sun et al., 2024). Here, we specifically tested periodic (grid-like) firing and distinguished it from boundary-vector cells (BVCs), corner cells (CCs), and place cells (PCs) using the procedures.

### Grid analyses and periodic firing analyses

Grid analyses were implemented following similar criteria to previous studies (Hafting et al., 2005). Spatial autocorrelograms were calculated for each firing map.


$$\begin{array}{l} r(\tau_{x},\tau_{y})=\\ \frac{n\sum \lambda (x,y)\lambda (x-\tau_{x},y-\tau_{y})-\sum \lambda (x,y)\sum \lambda (x,y)\sum \lambda (x-\tau_{x},y-\tau_{y})}{\sqrt{n\sum \lambda {\left(x,y\right)}^{2}-{\left(\sum \lambda (x,y)\right)}^{2}}\sqrt{n\sum \lambda {\left(x-\tau_{x},y-\tau_{y}\right)}^{2}}-\sum \lambda (x-\tau_{x},y-\tau_{y}))2} \end{array}$$

where r is the autocorrelation between the original firing rate map and its spatially shifted copy. The lag *τ* is normally one-dimensional for signal analysis. Because of the two-dimensional nature of the firing rate map, the autocorrelation has lags in the x- and y-directions: τx and τy. n is equal to the number of pixels. To determine the existence of a hexagonal grid firing structure, grid index, size, and orientation were calculated as described previously (Hafting et al., 2005; Brotons-Mas et al., 2017).

To obtain the “gridness index,” we extracted a circular portion of the spatial autocorrelogram generated from the spatial firing matrix. This circle was centered on the central peak, which was eliminated from the matrix. Then, we correlated the rotated versions, in steps of 6°, of this matrix with the original spatial autocorrelogram. The degree of spatial periodicity (“gridness” or “grid scores”) was determined for each recorded cell, and the grid index was calculated as previously described (Hafting et al., 2005; Sargolini et al., 2006).

Additionally, to further investigate the presence of periodic firing, we obtained the sum of values of the 2D resulting map in all its orientations, and we displayed the preferred orientation of these neurons in space as a polar plot (Krupic et al., 2012). These values were then plotted against the polar plot of the shuffled original firing map, *n* = 1,000. Only neurons that showed higher values than this confidence interval were considered as periodic neurons.

### Boundary-vector cells and corner cells

To identify BVCs, we followed the same methodology as previously described (Muessig et al., 2024). This analysis implied the comparison of the original map with the idealized BVC. Spatial shuffling was performed as described above. Model BVCs were fitted to these spatially shuffled rate maps as described above. To be classified as a BVC, the r_(max)_ value for each cell had to exceed the 95th percentile of the 1,000 shuffled r_(max)_ values generated for that specific cell and trial, while also meeting the same criteria for spatial modulation.

Corner cells were identified following the same procedure as described by Sun et al. (2024). The field location was taken as the regional maximum (x,y) of each region. Centroid and corner coordinates were detected and manually verified. For each field, we computed a corner score


corner score=(d1−d2)/(d1+d2)

where *d1* is the distance from the field maximum to the environment centroid, and *d2* is the distance to the nearest corner. The score ranges from −1 (centroid) to +1 (ideal corner). The total corner score of the cell was calculated as the mean of the corner scores across its firing fields. A neuron was classified as a corner cell only if its total index exceeded the 95th percentile of the shuffled distribution and if it had more than one firing field. Units that did not meet GC, BVC, or CC were deemed as place cells (PCs).

### Histological analysis

After completing the experiments, animals were euthanized with sodium pentobarbital (180 mg/kg at a concentration of 100 mg/mL) and transcardially perfused with saline, followed by 4% paraformaldehyde. This procedure was authorized by the Ethics Commission in accordance with current regulations and following veterinary recommendations. The brains were removed, sliced into 40-μm sections, and prepared for immunohistochemistry. For primary antibodies, we used anti-NeuN (1:500; Millipore #MAB377) and GFAP (1:1000; Merck SAB2500462-100UG; host: goat). For secondary antibodies, we used anti-goat (1:1000; Invitrogen A11055) for electrode localization. Cell nuclei were stained with DAPI (Sigma-Aldrich, D9542-10ML). Electrode tracks were identified when visible, and their positions were estimated by comparison with anatomical landmarks using the Paxinos and Franklin stereotaxic atlas. In addition, the presence of local electrophysiological signatures (e.g., ripple activity and theta oscillations) provided further confirmation of electrode placement. In those cases, where a clear electrode track was not detectable, the location of the electrodes was inferred based on implant coordinates, electrode design, and characteristic electrophysiological landmarks.

### Statistical analysis

Distribution of dependent variables was tested for normality and for equal variance. Parametric statistical tests were used for normally distributed variables, and non-parametric tests were used for variables that did not show normal distribution. All descriptive values are expressed as mean ± the standard error (S. E.). Statistical significance was set at *p* < 0.05. All statistical analyses were performed using SPSS (IBM, v27) and the MATLAB Statistics Toolbox (Mathworks, Natick, MA, United States).

## Results

### Tetrode recordings in the CA1 and SUB

To investigate spatially periodic cells in subiculum, we conducted recordings in standard (50 × 50 cm) and large (70 × 70 cm) open-field arenas. Across nine mice—five implanted in CA1, one in SUB, and three in both regions—histological analysis confirmed the placement of three tetrodes in three different mice in the SUB (Figures 1A,B). In total, we recorded 147 neurons from CA1 and 65 neurons from the SUB. Units were classified based on spike duration, waveform shape, and firing rate (Figure 1C), following established criteria (Sharp and Green, 1994). Of these, 74 CA1 neurons and 28 subicular cells met the pyramidal and spatial-modulation criteria (Methods).

Unit isolation quality did not differ between regions (overlap probability: CA1, 0.06 ± 0.01; SUB, 0.10 ± 0.02; Mann–Whitney U = 4,290, *p* = 0.236), consistent with previous reports (Del Pino et al., 2017; Hill et al., 2011). Spike amplitude and duration were also similar (spike duration: 477.5 ± 7.8 μs vs. 491.0 ± 10.4 μs, U = 862, *p* = 0.192; spike amplitude: 254.1 ± 11.9 μV vs. 226.1 ± 15.6 μV, U = 943, *p* = 0.48; Figures 1D,E). In contrast, subicular neurons fired faster than CA1 neurons (mean rate: 1.8 ± 0.2 Hz vs. 1.2 ± 0.12 Hz; U = 745, *p* = 0.02; Figure 1F), consistent with previous reports (Sharp and Green, 1994; Brotons-Mas et al., 2010; Mizuseki and Kitanishi, 2022).

The interaction between the CA1 and the SUB plays a critical role in organizing the input–output dynamics within the hippocampal formation. This dialog depends on the specific timing of the activity of different populations of neurons in different theta cycle windows (Valero and de la Prida, 2018). Therefore, examining the relationship between CA1 and subicular firing and local LFP is fundamental to understanding the temporal dynamics of the hippocampal formation. We investigated the relationship between the firing of CA1 and subicular neurons with the locally recorded theta (4-12 Hz). To target the pyramidal cell layer, we selected tetrodes on which ripples showed no slow (positive or negative) component, a standard indicator of pyramidal-layer placement. In CA1, preferred firing phases were bimodally distributed, with units clustering around the ascending and descending phases of the local theta cycle and an additional concentration near the trough (Figure 1G). For subiculum, we applied the same tetrode-selection criterion and computed phase from the local LFP on the same tetrode; subicular neurons preferentially fired near the theta peak in the tetrode database (Figure 1H).

### A group of subicular neurons showed grid and spatially periodic firing

**Figure 2:**
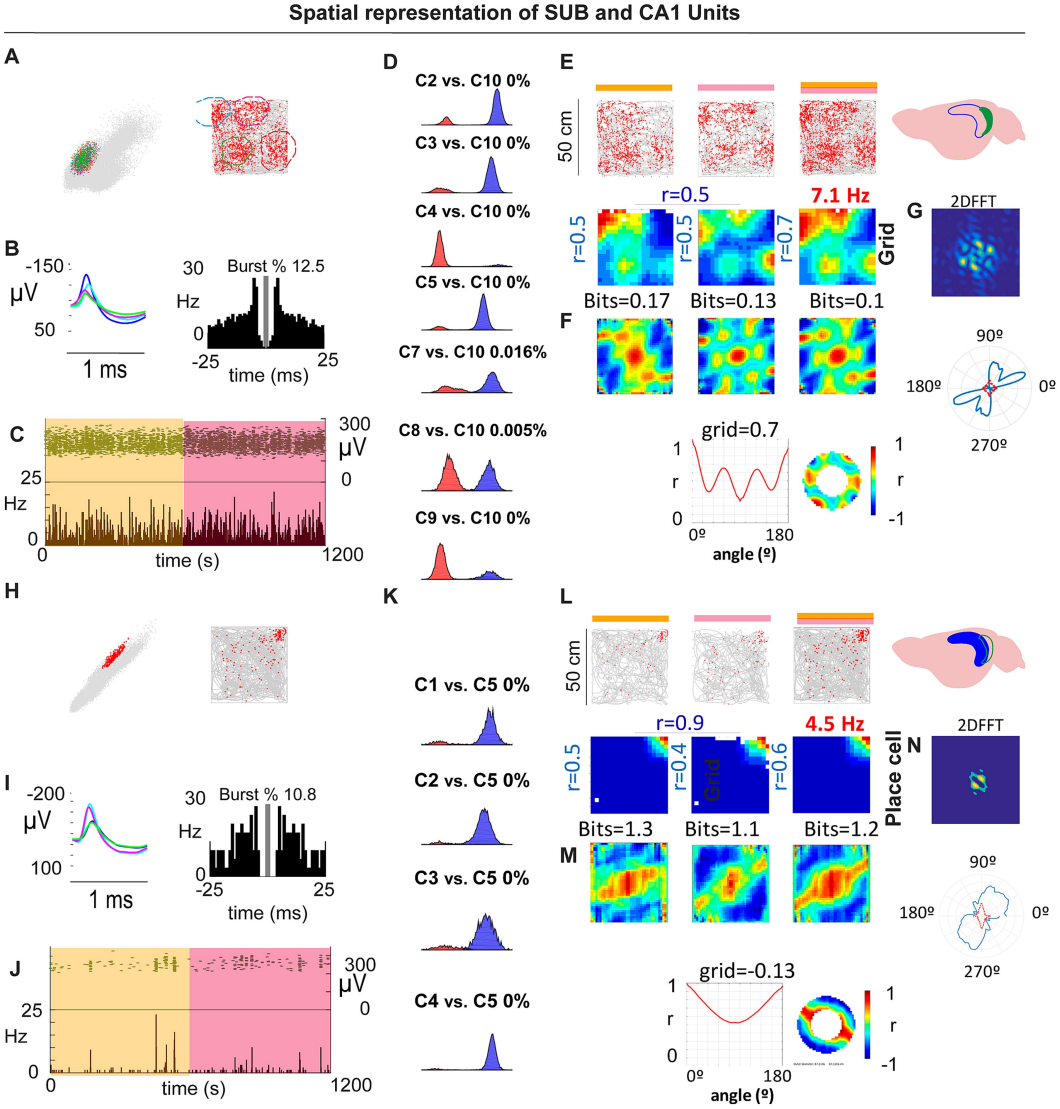
**(A–C)** Example SUB periodic cell: cluster isolation, waveform/ISI with burst %, and stable firing across the session. **(D)** Overlap probability controls for isolation quality (example comparisons). **(E,F)** Rate maps and autocorrelograms across spatial bins with corresponding gridness/periodicity summaries and 2D FFT **(G)**. **(H–J)** Example CA1 place cell recorded in the same arena: isolation **(K)**, rate maps/autocorrelograms **(L,M)** and 2D FFT **(N)**. CA1 pyramidal units did not exhibit periodic/grid signatures under these conditions.

In rats, subicular spatial coding comprises a heterogeneous mix of place cells (PCs) and boundary-vector cells (BVCs), as well as corner cells (CCs; Lever et al., 2009; Brotons-Mas et al., 2017; Sun et al., 2024). Given the larger body size of rats relative to typical arena dimensions, spatial periodicity may have been difficult to detect. In mice recorded in 50 × 50 cm arenas, we observed that several subicular neurons exhibited spatial periodic firing resembling a grid-like structure. In contrast to previous reports (Brotons-Mas et al., 2017), these units had pyramidal-like waveforms, were stable within a session, and were well isolated (Figures 2A–D). Their spatial firing showed hexagonal periodicity by both autocorrelogram and frequency-domain analyses (Figures 2E–G). CA1 pyramidal neurons recorded in the same arenas did not show comparable periodic patterns (Figures 2H–N). Overall, 4 of 28 spatially modulated subicular units (14.3%) met our gridness criterion (three cells in one mouse and one cell in a second mouse).

**Figure 3:**
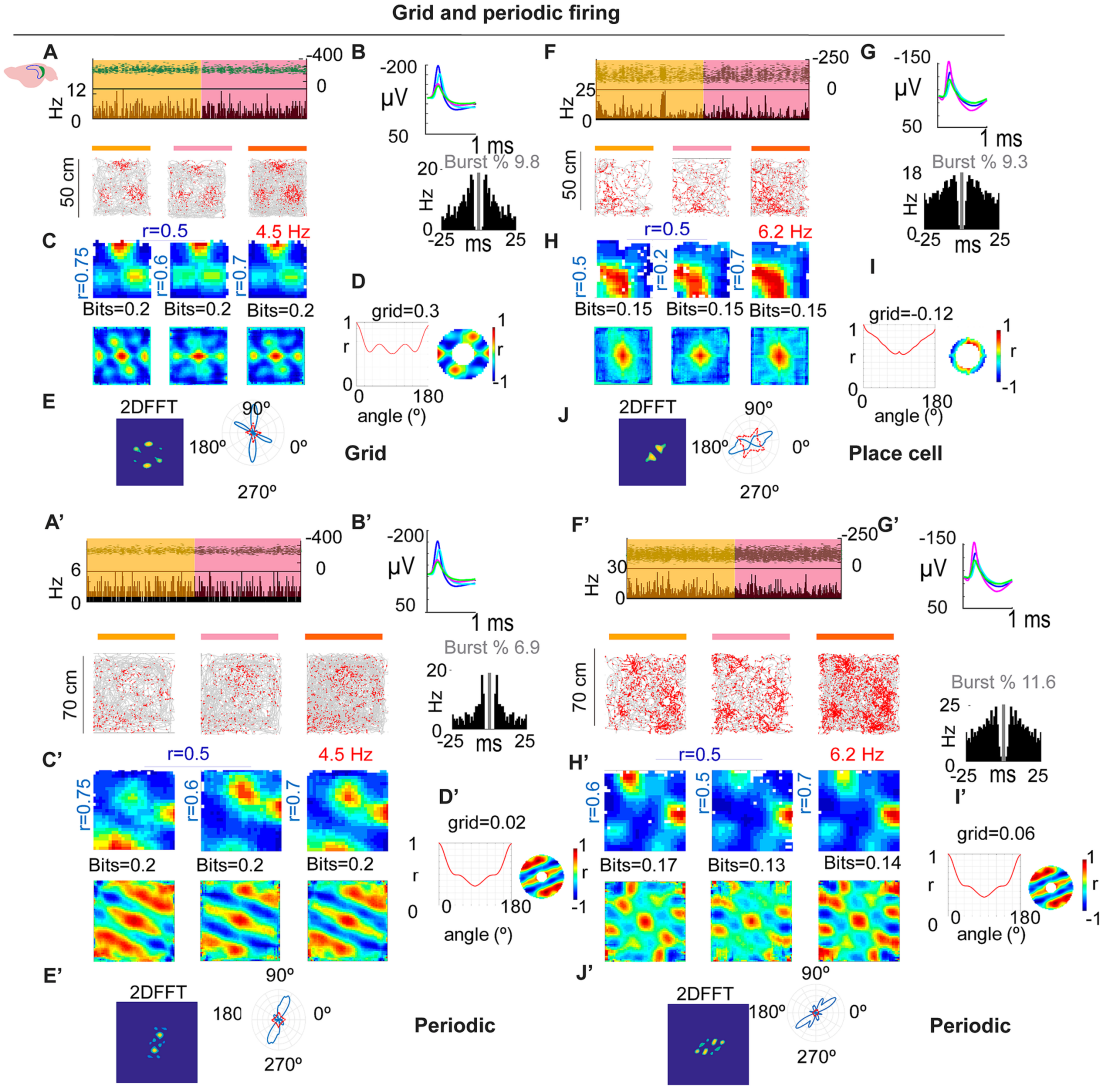
Grid and spatially periodic firing across arena size. **(A–D)** Example SUB neuron in 50 × 50 cm: stable firing **(A)**, waveforms/ISI **(B)**, rate maps and autocorrelograms **(C)**, grid/periodicity summary **(D)** with 2D FFT **(E)**. **(A′–E′)** Same neuron in 70 × 70 cm: periodic structure persists but deviates from perfect hexagonal symmetry (see main text). **(F–J,F′–J′)** Second example showing a place-like pattern in the small arena **(G)** that becomes periodic in the large arena, as confirmed by the 2D FFT **(J,J′)**.

To test the robustness of subicular periodicity across scales, we tracked individual SUB neurons across 50 × 50 cm and 70 × 70 cm boxes. Some cells deviated from perfect hexagonal symmetry in the larger arena yet retained clear periodic structure, similar to observations in MEC under certain conditions (Krupic et al., 2012; Figures 3A–E,A’–E’). Conversely, we identified at least one cell that showed a single dominant place field in the 50 × 50 cm arena, but developed periodic firing in the 70 × 70 cm arena, as revealed by the 2D FFT and related metrics (Figures 3F–J,F’,J’).

### Spatial modulation of SUB neurons was lower than that of CA1 neurons

**Figure 4:**
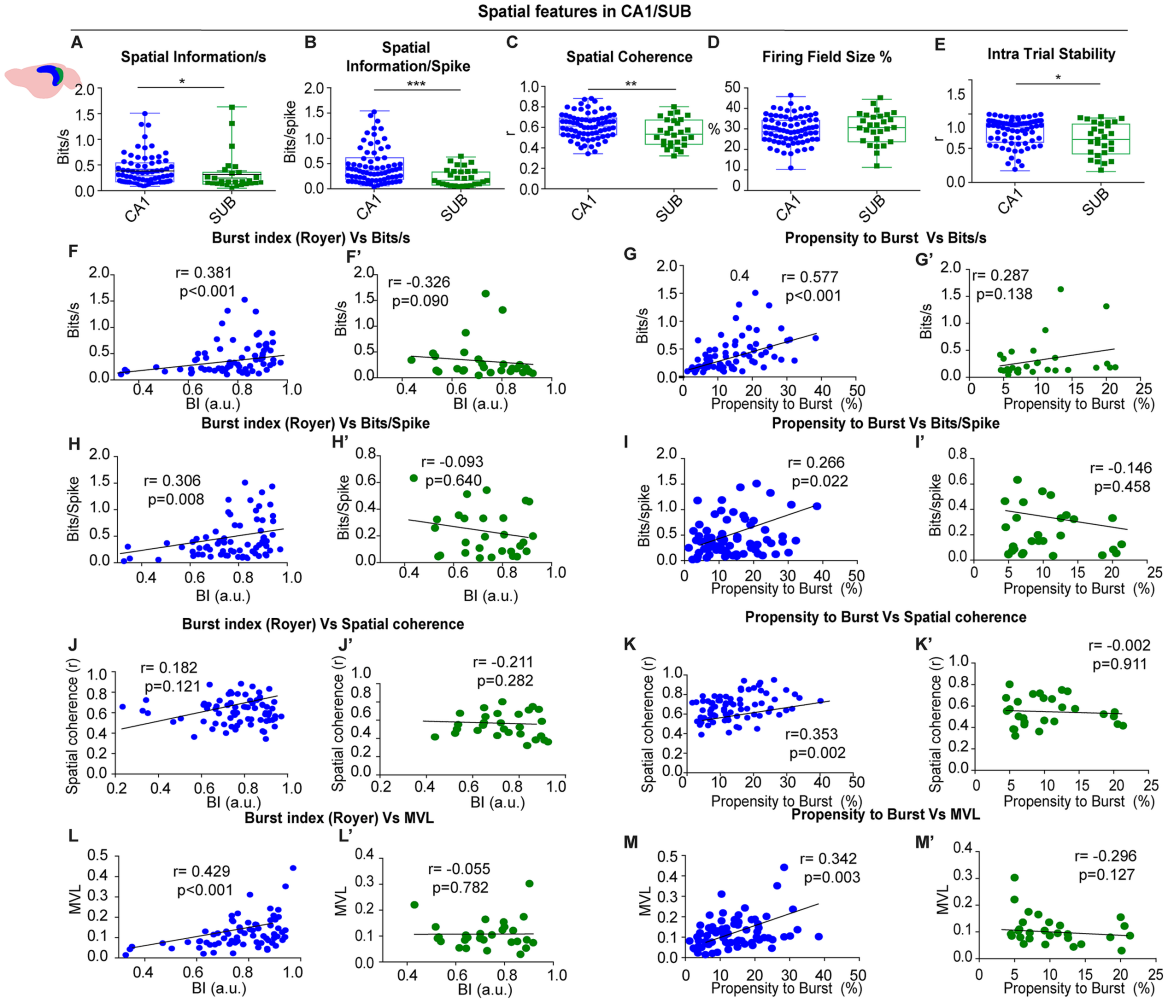
Spatial features in CA1 vs. SUB **(A–E)** group comparisons: spatial information per second, spatial information per spike, spatial coherence, firing-field size and intra-trial stability were higher in CA1 **(F–M′)** correlations between burst index or propensity to burst and spatial metrics (bits/s, bits/spike, coherence, MVL) for CA1 (blue) and SUB (green).

Spatial coding differs across hippocampal subregions (Barnes et al., 1990; Oliva et al., 2016). We compared CA1 and SUB using standard metrics—spatial information per second, spatial information per spike, spatial coherence, and firing-field size. We found significant differences between CA1 and subicular neurons for the spatial information per second, spatial information per spike, and for the spatial coherence, consistent with previous reports (Figures 4A–C). In contrast, (Figure 4D), no significant differences were observed for the firing field size (CA1 vs. SUB: bits/s 0.39 ± 0.01 vs. 0.322 ± 0.06 μs: U = 738.0, *p* = 0.025; bits/spike 0.43 ± 0.04 vs. 0.32 ± 0.06: U = 611.0, *p* = 0.001, Mann–Whitney; spatial coherence: (r), 0.61 ± 0.01 vs. 0.54 ± 0.02: t_(100) =_ 2.442, *p* = 0.016; firing field size (%), 29.02 ± 0.7 vs. 30.0 ± 1.14, t_(100) =_ -0.634, *p* = 0.528).

Because temporal instability can mimic rate remapping and obscure periodicity (Leutgeb et al., 2005), we assessed within-session stability by splitting each session into two 10-min halves (Figure 4E) and by computing bin-by-bin spatial correlations. We found to eliminate this sentence that place cells in the CA1 region showed significantly higher spatial consistency (CA1 vs. SUB: intra-trial stability coherence (r), 0.72 ± 0.02 vs. 0.61 ± 0.04: U = 764.0, *p* = 0.041, Mann–Whitney). However, periodic neurons showed high stability within each trial (See Figure 3 for different examples), ruling out remapping effects as a confounding factor in the identification of spatially periodic firing.

### Spatial modulation of SUB units in the proximal region was not determined by bursting activity

The spatial information encoding is closely linked to the electrophysiological characteristics of hippocampal pyramidal neurons. Specifically, place cells are known to exhibit complex spikes, or bursting activity, which is characterized by the generation of trains of action potentials within an envelope of 15–20 ms. This bursting pattern is thought to enhance the fidelity of spatial information across different brain regions (Simonnet and Brecht, 2019). We investigated whether there was a specific relationship between bursting patterns and the spatial modulation in the CA1/SUB axis.

We found that in CA1 bursting metrics correlated with spatial modulation (Figures 4F–M). The burst index (BI) correlated with information per second (*r* = 0.381, *p* < 0.001, Spearman) and information per spike (*r* = 0.306, *p* = 0.008), showed a trend for spatial coherence (*r* = 0.182, *p* = 0.18, Spearman), and was strongly related to theta locking (MVL; *r* = 0.429, *p* < 0.001, Spearman). The propensity to burst (PrtB) showed a similar pattern: bits/s (*r* = 0.577, *p* < 0.001), bits/spike (*r* = 0.266, *p* = 0.022, Spearman), spatial coherence (*r* = 0.353, *p* = 0.002, Spearman), and MVL (*r* = 0.342, *p* = 0.003, Spearman). These relationships indicate that stronger bursting is associated with stronger spatial coding and tighter theta-phase locking in CA1. Remarkably, we observed no correlation between bursting properties and spatial modulation in SUB-recorded units, (Figures 4F’–M’; BI vs. bits per second, *r* = −0.326, *p* = 0.09; BI vs. bits per spike *r* = −0.093, *p* = 0.640; BI vs. spatial coherence, *r* = −0.211, *p* = 0.282; BI vs. mean vector length, *r* = −0.055, *p* = 0.782, Spearman). A similar pattern was observed when we compared the propensity to burst with the spatial modulation (PrtB vs. bits per second, *r* = 0.287, *p* = 0.138; PrtB vs. bits per spike *r* = −0.146, *p* = 0.458; PrtB vs. spatial coherence, *r* = −0.002, *p* = 0.991; PrtB vs. mean vector length, *r* = −0.296, *p* = 0.127, Spearman).

This indicates that CA1 neurons with a higher tendency to burst exhibited stronger spatial modulation, whereas in subicular neurons, bursting activity did not predict spatial modulation, whether periodic or non-periodic.

### Multisite recordings

**Figure 5:**
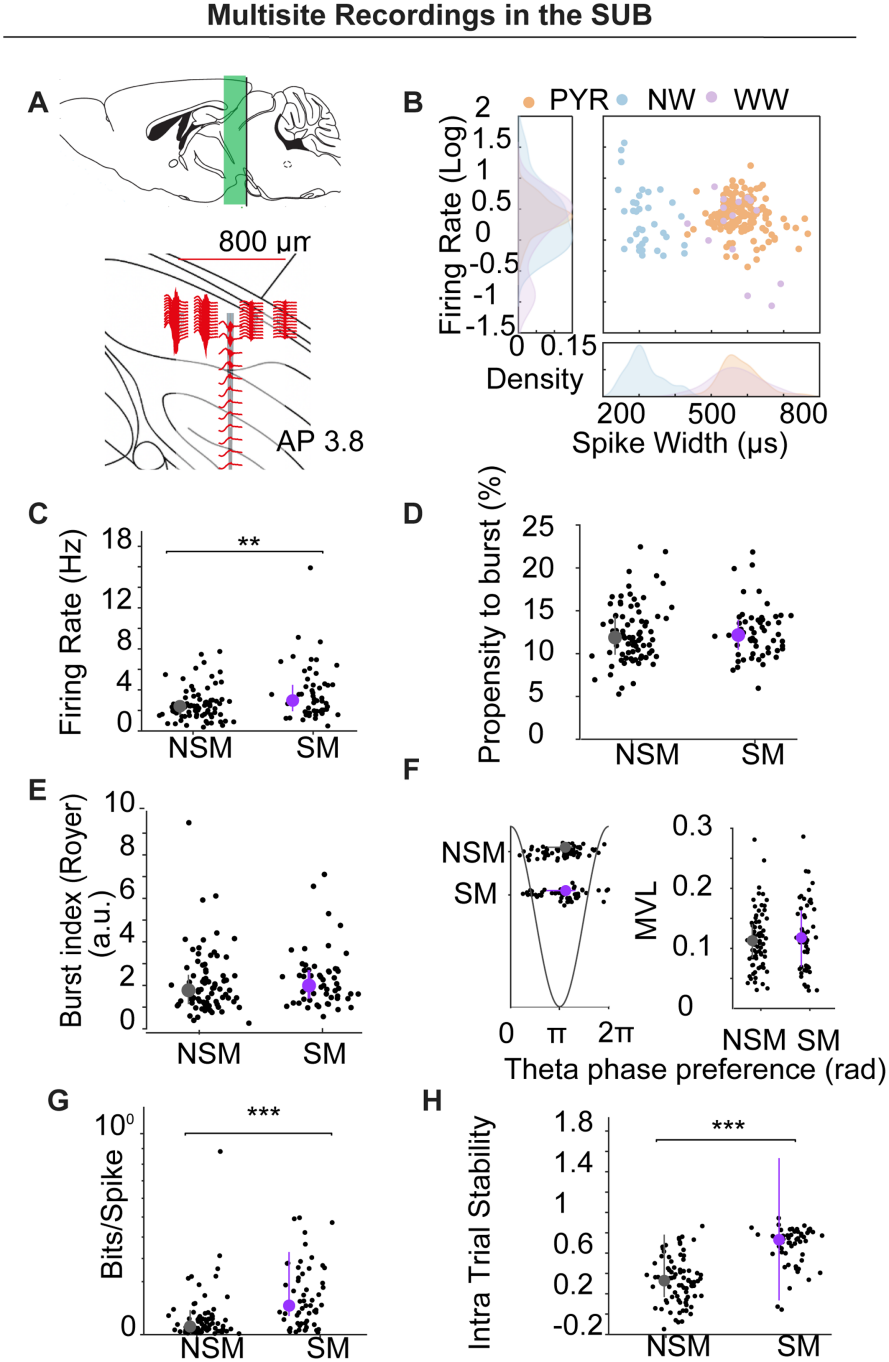
Multisite silicon-probe recordings in subiculum. **(A)** Schematic of probe geometry and dorsoventral span (~800 μm) targeting dorsal SUB (AP − 3.8). **(B)** Cell-type classification by spike width and firing rate: pyramidal (PYR, orange), narrow-waveform interneurons (NW, blue), wide-waveform interneurons (WW, purple); density plots shown at margins. **(C–H)** Comparisons for firing rate **(C)**, propensity to burst **(D)**, burst index **(E)**, theta-phase preference/locking **(F)**, spatial information (bits/spike) **(G)**, and intra-trial stability **(H)** between NSM and SM pyramidal neurons. SM fired faster, had higher bits/spike and spatial stability with no differences in PrtB, BI, or theta metrics.

To validate and extend the tetrode findings, we implanted two additional mice with 64-channel silicon probes targeting dorsal subiculum (see Figure 5A). Data were sorted using KiloSort2 and manually curated in Phy2, with cell-type labels assigned via CellExplorer (Petersen et al., 2021; see Methods section). We isolated 196 units with clear refractory periods: pyramidal neurons (PYR, *n* = 142), narrow-waveform interneurons (NW, *n* = 35), and wide-waveform interneurons (WW, *n* = 15; see Figure 5B). Four units were unclassified.

### Spatial modulation

A unit was classified as spatially modulated (SM) if its rate-map spatial coherence exceeded the 95th percentile of a 1,000-iteration time-shift shuffle (See Methods). Overall, 67/196 units (34.2%) met this criterion. Unless otherwise noted, the analyses below are restricted to pyramidal neurons.

Firing rates were higher in SM pyramidal neurons than in non-spatially modulated (NSM) neurons (3.672 ± 0.347 Hz vs. 2.585 ± 0.173 Hz; U = 4,967, *p* = 0.0065; Mann–Whitney; Figure 5C). By contrast, burst metrics—propensity to burst (PrtB) and burst index (BI)—and theta-phase preference/locking did not differ between SM and NSM (PrtB: 2.441 ± 0.399 vs. 12.389 ± 0.377; U = 5,545, *p* = 0.849; Mann–Whitney; Figure 5D; BI: 2.286 ± 0.174 vs. 2.081 ± 0.159; U = 5,251, *p* = 0.139; Mann–Whitney, Figure 5E); Theta phase (mean ± circSD, 3.443 ± 1.086 vs. 3.424 ± 0.978, Watson–Williams test: F (1) = 0.007, *p* = 0.935; Theta MVL: 0.114 ± 0.008 vs. 0.120 ± 0.006, U test: U = 4,879, *p* = 0.524; Mann–Whitney; Figure 5F). As expected, SM units showed greater spatial information and within-session (intra-trial) stability than NSM (bits/spike: 0.145 ± 0.017 vs. 0.062 ± 0.012, *U* = 4,469, *p* < 0.0001; stability: 0.672 ± 0.025 vs. 0.329 ± 0.025, *U* = 3,877, *p* < 0.0001; Figures 5G,H).

### Incidence of periodic cells and other spatial classes in multisite recordings

**Figure 6:**
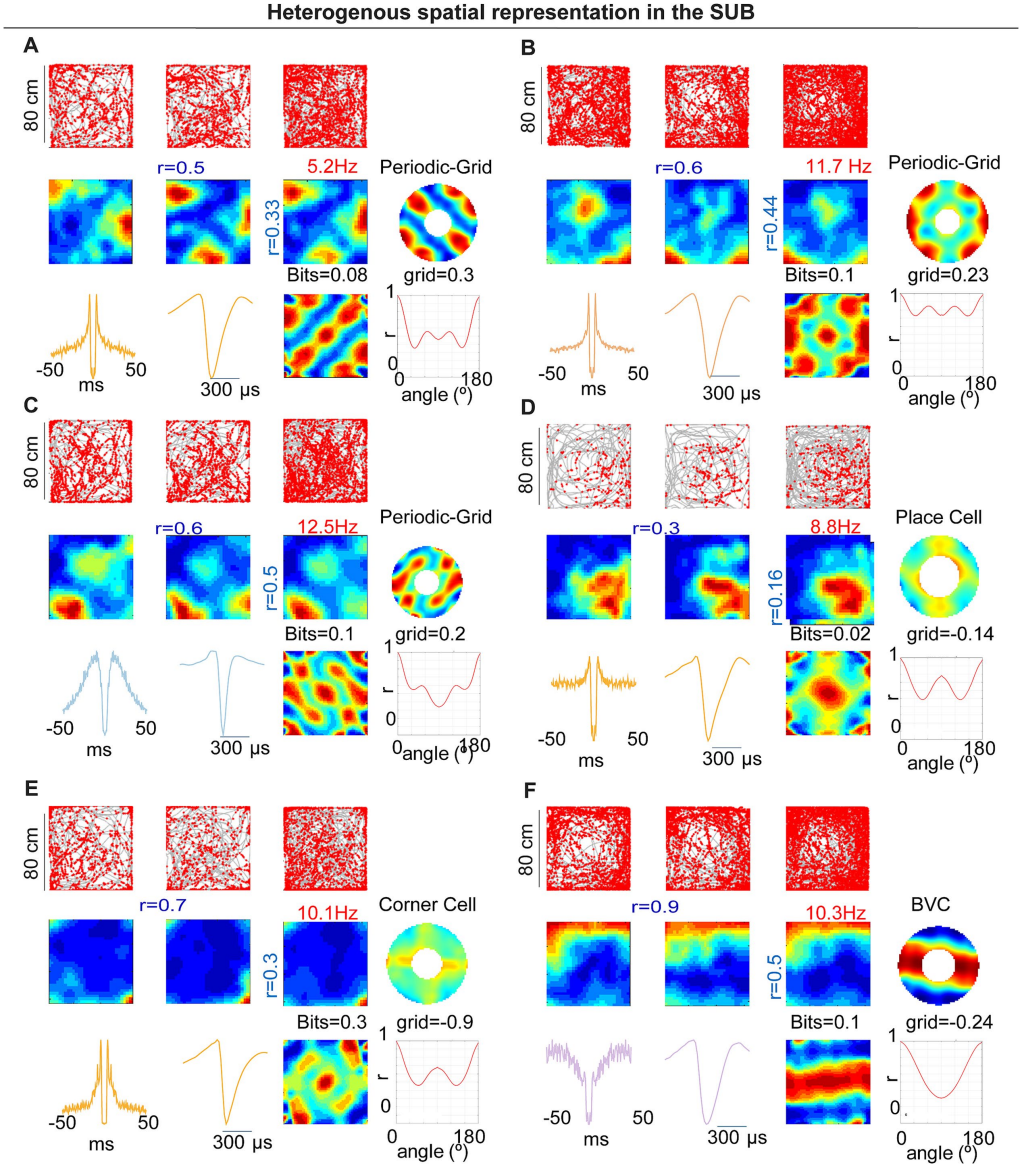
Heterogeneous spatial representations in subiculum (probe cohort, 80 × 80 cm). **(A–C)** Examples of periodic/grid-like neurons: trajectories with spikes, rate maps across time segments, spatial autocorrelograms, periodicity summaries (polar/orientation energy), and spike waveforms/ISI. **(D)** Example place cell. **(E)** Example corner cell with a high corner-score field. **(F)** Example boundary-vector cell (BVC). For each neuron, grid score/periodicity measures and bit values are indicated on the panels. Across the cohort, spatial classes (PC, BVC, CC, GC, and NSM) were distributed along the proximodistal axis.

In the probe cohort, 7/67 SM units (10.4%) met our *a priori* grid/periodicity criteria (four and three cells in the two animals, respectively; Figures 6A–C). Five of these were PYR and two were NW interneurons (Figure 6C). This incidence closely matches the tetrode dataset (4/28 = 14.3%), under identical detection and shuffling thresholds, indicating that periodic firing in mouse subiculum replicates across animals and recording methods. We also identified place cells (PCs; 36 of 67, 53.7%), boundary-vector cells (BVCs; 19 of 67, 28.4%), and corner cells (CCs; 5 of 67, 7.5%; Figures 6D–F).

**Figure 7:**
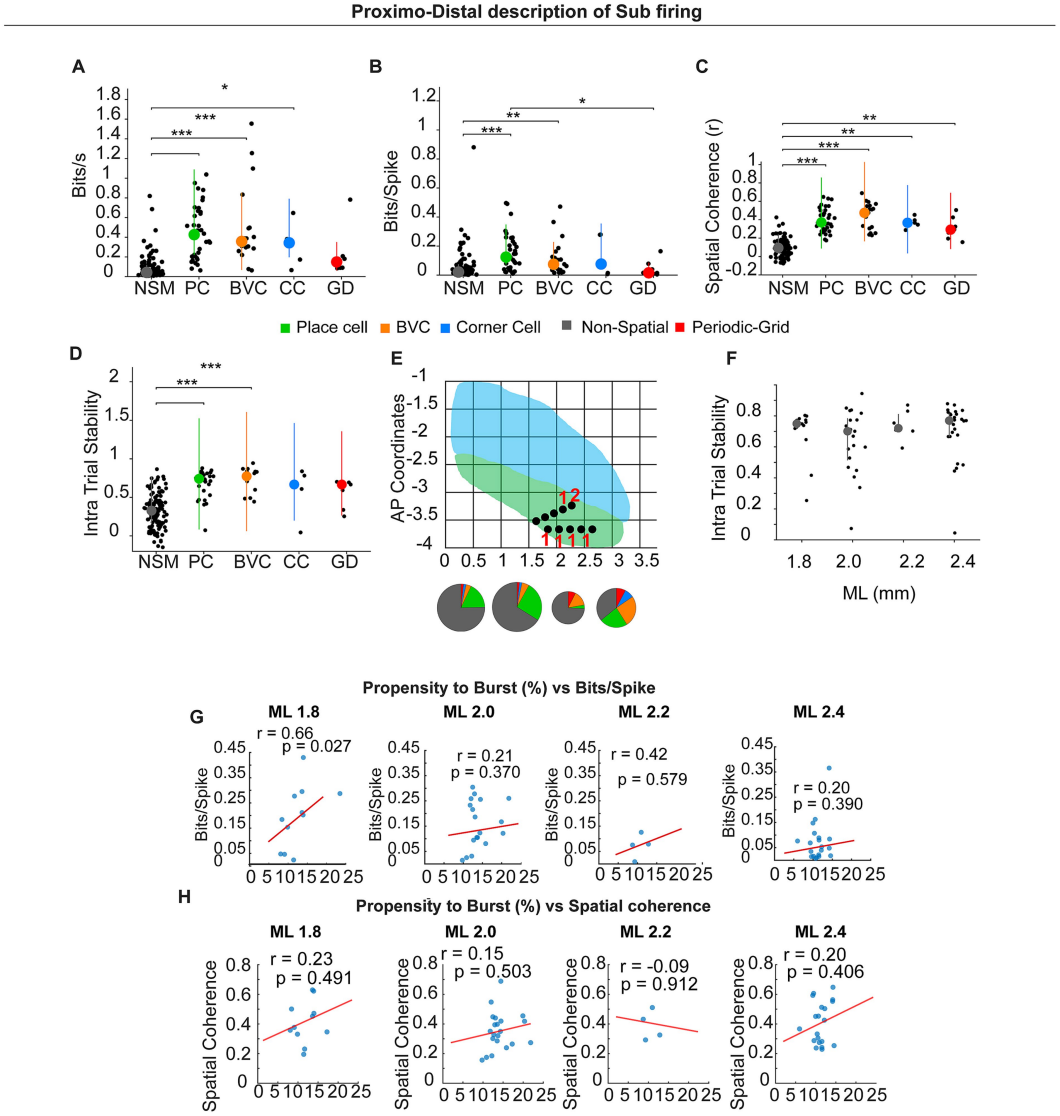
Different spatial types in the sub and Proximo–distal description of subicular firing. **(A–D)** Summary of spatial metrics across cell classes: NSM (non-spatial), PC (place cell), BVC (boundary-vector cell), CC (corner cell), and GD (grid/periodic). **(E)** Schematic of dorsal subiculum showing mediolateral (ML) and anteroposterior (AP) CA1 (blue) SUB (green). Black dots indicate recording sites; pie charts show cell-type composition within each ML bin representing numbers giving in the result text. **(F)**. Intra-trial stability across the proximo-distal axis. **(G,H)** Correlations (spatially modulated pyramidal neurons only) between propensity and burst (%) and spatial information (bits/spike) **(G)** or spatial coherence **(H)** computed within ML bins (1.8, 2.0, 2.2, 2.4 mm). Lines show least-squares fit; r and *p* values are Pearson correlation coefficients. A significant positive relation appears distally (ML 1.8: *r* = 0.66, *p* = 0.027); other anatomical locations show no significant correlation.

We next investigated potential differences across neuron types across multiple parameters; for this analysis, all neuron types were included regardless of their electrophysiological type (Figures 7A–D). Spatial information per second was higher in PC, BVC, and CC than in NSM (NSM, 0.080 ± 0.011; PC, 0.449 ± 0.046; BVC, 0.497 ± 0.092; CC, 0.322 ± 0.099; GC, 0.226 ± 0.094; χ^2^_(4)_ = 96.523, *p* < 0.0001, Kruskal–Wallis test; *post-hoc* Dunn–Holm: PC > NSM, adjusted *p* < 0.0001; BVC > NSM, adjusted *p* < 0.0001; CC > NSM, adjusted *p* = 0.035). Spatial information per spike also differed among classes (NSM, 0.052 ± 0.009; PC, 0.156 ± 0.021; BVC, 0.121 ± 0.029; CC, 0.131 ± 0.061; GC, 0.047 ± 0.022; χ^2^_(4)_ = 42.985, *p* < 0.0001, Kruskal–Wallis test; *post-hoc*: PC > NSM, adjusted *p* < 0.0001; BVC > NSM, adjusted *p* = 0.004; PC > GC, adjusted *p* = 0.031). Spatial coherence was higher for all spatial classes compared with NSM (NSM, 0.095 ± 0.008; PC, 0.392 ± 0.021; BVC, 0.441 ± 0.033; CC, 0.369 ± 0.027; GC, 0.308 ± 0.047; χ^2^_(4)_ = 116.918, *p* < 0.0001; Kruskal–Wallis test; *post-hoc*: PC > NSM, adjusted *p* < 0.0001; BVC > NSM, adjusted *p* < 0.0001; CC > NSM, adjusted *p* = 0.003; GD > NSM, adjusted *p* = 0.005).

We also verified that there were no significant differences in intra-trial spatial stability between spatial types. On the other hand, they presented higher spatial stability than non-spatially modulated neurons, although this effect was significant only for PC and BVC (NSM, *r* = 0.3199 ± 0.02; PC, *r* = 0.68 ± 0.02; BVC, *r* = 0.74 ± 0.03; CC, *r* = 0.58 ± 0.14; GC, *r* = 0.56 ± 0.07; χ^2^_(4)_ = 84.93, *p* > 0.0001, Kruskal–Wallis test; *post-hoc*: PC > NSM, adjusted *p* < 0.0001; BVC > NSM, adjusted *p* < 0.0001; CC vs. NSM, adjusted *p* = 0.161; GC vs. NSM, adjusted *p* = 0.250). Spatial cell types occurred throughout the sampled proximodistal span (Figure 7E).

### Proximodistal axis spatial stability and bursting–spatial coupling

We investigated whether intra-trial spatial stability differed along the proximodistal axis. For this analysis, we included all spatially modulated neurons, independent of their electrophysiological profile. We found no significant differences in these parameters (Spike duration ms 1.8 mm: 0.6920 ± 0.0426; 2.0 mm: 0.6383 ± 0.0451; 2.2 mm: 0.7397 ± 0.0345; 2.4 mm: 0.6988 ± 0.0375 χ^2^(3) = 2.148, *p* = 0.542, Kruskal–Wallis test). Similar results were obtained for only pyramidal neurons (data not shown).

In the tetrode dataset—which predominantly sampled proximal dorsal subiculum—burst metrics (BI, PrtB) did not correlate with spatial information. Considering the proximal-to-distal gradient in bursting propensity, we re-examined this relationship in the probe dataset, which sampled ~800 μm along the proximodistal axis (Figures 7G,H). Considering spatially modulated (pyramidal only) neurons, bits/spike correlated positively with burst propensity in the most distal bin (ML 1.8: *r* = 0.66, *p* = 0.027), but not in more proximal bins (ML 2.0: *r* = 0.21, *p* = 0.370; ML 2.2: *r* = 0.42, *p* = 0.579; ML 2.4: *r* = 0.20, *p* = 0.390).

## Discussion

We provide the first evidence in mice that subicular pyramidal neurons exhibit grid-like/spatially periodic firing, using two independent technologies—custom tetrodes and high-density linear silicon probes. In the probe cohort, 7 of 67 spatially modulated cells (10.4%) met our grid/periodicity criteria (5 pyramidal and 2 interneurons), an incidence that closely matched the tetrode dataset (4/28 = 14.3%), indicating robustness and replicability across methods. Unit isolation met established sorting-quality benchmarks (Krupic et al., 2012; Hill et al., 2011; Navratilova et al., 2016), and isolation quality did not differ between CA1 and SUB. Finally, spike waveforms and firing statistics were consistent with pyramidal neurons and inconsistent with putative axonal recordings (Robbins et al., 2013).

Multisite (silicon-probe) recordings yielded higher unit counts and broader proximodistal coverage within dorsal SUB, enabling identification of multiple spatial classes—place cells, boundary-vector cells, and corner cells—all present across the proximodistal axis, and to better understand how bursting and firing rate might contribute to spatial information transmission.

### SUB spatial representation is different from CA1

We observed that PCs in the CA1 showed higher spatial resolution as compared to subicular cells. This apparent lower spatial modulation of subicular neurons is related to their higher firing rate. Such augmented activity seems to serve as a mechanism to improve information routing from the hippocampal formation to other brain regions (Kitanishi et al., 2021).

In the tetrode dataset, subicular neurons tended to fire near the center/peak of the theta cycle, which contrasts with prior reports showing a bias toward the descending phase and earlier than CA1 (Kitanishi et al., 2021). This discrepancy may reflect recording location across the deep–superficial axis sampling. Consistent with this interpretation, the multisite probe recordings, which provide better laminar localization, revealed a phase preference like the published pattern (Kitanishi et al., 2021). Thus, the apparent mismatch in the tetrode data likely stems from limited control over laminar position.

### Proximodistal organization in the subiculum

Inputs to the SUB are topographically organized, and in this way, the proximal region of the SUB receives information about inputs from LEC, known to be involved in the temporal coding of episodes (Tsao et al., 2018), while more distal regions integrate inputs from the MEC involved in spatial coding (Hafting et al., 2005), see O’Mara et al. for a comprehensive review (O’Mara et al., 2009; O’Mara et al., 2001). In addition, there is a proximodistal gradient of burstiness, from lower in the proximal to higher in the distal SUB, that might indicate a segregation of functions (Cembrowski et al., 2018).

Our multisite recordings show that the dorsal subiculum hosts a diverse ensemble of spatial codes rather than a single canonical type. Within the same animals, we observed place cells (PCs), boundary-vector cells (BVCs) that anchor firing to the geometry of nearby boundaries, corner cells (CCs) with fields concentrated near vertices, and a subset of neurons with spatially periodic (grid-like) activity. Importantly, these classes were distributed along the proximodistal axis, rather than confined to a mediolateral specific coordinate, indicating that geometric and periodic representations are the features of subicular circuitry. This heterogeneity suggests that SUB integrates inputs from CA1 and entorhinal cortices to form complementary codes—spatial, boundary-anchored (BVC/CC) and periodic tiling—that can support navigation and context generalization across environments.

The presence of these codes across the proximodistal extent also implies parallel output channels (O’Mara et al., 2009; O’Mara et al., 2001) from SUB to downstream cortico-subcortical targets, enabling these different signals to be differentially routed rather than relying on a single encoding scheme. This could be critical for the involvement of the SUB in different cognitive processes. In this sense, subicular neurons seem capable of maintaining active information during the inter-trial in a working memory task (Deadwyler and Hampson, 2004). Such sustained or modulatory activity could help signal the temporal structure of episodes, with distinct proximal–distal inputs contributing to the organization of different information streams.

In our tetrode recordings in the proximal SUB, spatial stability was lower than in CA1, consistent with stronger LEC influence. However, in the multisite dataset, we found no significant proximodistal differences in intra-trial stability, suggesting that stability per se is relatively uniform along the axis as well as across different spatial cell types (BVC/CC vs. periodic).

It is relevant to mention that, unlike CA1, where bursting often correlates with spatial information and is thought to enhance synaptic reliability, tetrode recordings in the subiculum did not show a uniform burst–spatial coupling. However, our multisite recorded data resolved this discrepancy: burst propensity correlates with spatial information only at more distal recording sites, whereas proximal SUB shows no such relationship. This proximodistal dissociation suggests two coding regimes within SUB. Proximally, spatial information is supported chiefly by elevated firing rates—a strategy that can improve signal-to-noise and downstream reliability through temporal averaging and redundant spiking. Distally, bursting contributes additional coding power beyond rate aligning distal SUB more closely with CA1-like burst–information coupling. Given the known gradients in intrinsic excitability, burst propensity, and projection targets along SUB (Cembrowski et al., 2018), these results are consistent with a functional multiplexing: rate-dominated coding proximally and burst-augmented coding distally. Such an organization could tailor information transfer to distinct downstream circuits, with bursts selectively engaging synapses or targets that are burst-sensitive, while higher rates maintain robust throughput where bursts are less impactful.

### The role of periodic firing in the SUB in spatial cognition

Speed and direction of movement are critical for path integration, and both inputs have been proposed as essential for grid cells. This could be provided by GC ensembles (Zutshi et al., 2017). From a mechanistic perspective, the computation of speed may rely on theta oscillations, which are strongly correlated with velocity, as well as on speed cells (Kramis et al., 1975; Chen et al., 2011; Long et al., 2014; Kropff et al., 2021; Abad-Pérez et al., 2023). Moreover, the direction of movement is of great relevance (Raudies et al., 2015). This directional input may be provided by axis cells described in the SUB (Olson et al., 2017). Complementary to these inputs, the speed and directional signals could also be shaped by periodic cells in the MEC, pre-parasubiculum and SUB units reported here (Olson et al., 2017; Jeewajee et al., 2008). The redundancy of different speed and direction inputs might provide a more robust spatial integration for the navigation across different conditions, for example, across light–dark transitions.

Our new evidence of spatially periodic cells suggests that the SUB may contribute to generating spatial representations by supporting periodic cells and GC in the pre-parasubiculum and MEC. This is supported by anatomical data that confirm the strong afferences sent by the SUB to the pre- and parasubiculum and the deep layers of the MEC (O’Mara et al., 2009; O’Mara et al., 2001). Additionally, the SUB might provide input from boundary vector cells (BVCs) and periodic neurons to hippocampal place cells through its back projection to the CA1 area (Xu et al., 2016). In this way, circuits involving SUB-MEC, SUB-CA1, might support the transformation of the grid produced by alterations of the geometry of space (Krupic et al., 2015). Finally, SUB spatial representation appears to depend on thalamic inputs and might mediate changes in GC due to thalamic lesions, also known to be relevant for GC in MEC (Frost et al., n.d.).

In addition, two interneurons also met our grid/periodicity criteria, indicating that inhibition can participate directly in spatially structured coding. This is consistent with a recent publication (Valero et al., 2025), demonstrating a cooperative role for interneurons in shaping spatial representations.

### Data limitations and context of the results

One limitation of the tetrode cohort was the small N, with grid-like firing observed in two mice. Nevertheless, those units were stable across sessions and met stringent isolation criteria (refractory-period violations and cluster-overlap probability). In one implant, histology was incomplete, and the tetrode tip was not unambiguously visualized; depth was inferred from robust ripple activity, raising the possibility of partial sampling of adjacent regions (e.g., presubiculum), where ripples also occur (Chrobak and Buzsáki, 1996). However, we obtained only one neuron from this implant. To address these concerns, we added an independent multisite probe cohort that provided higher yield and proximodistal coverage. In this dataset, 7 of 67 spatially modulated units (10.4%) met our *a priori* periodic/grid criteria (5 pyramidal and 2 interneurons), an incidence that closely matched the tetrode dataset (4/28 = 14.3%) under identical analysis and shuffle thresholds. The replication across technologies and animals, together with consistent quality criteria and cell-type classification, substantially mitigates the small-sample caveat and supports the conclusion that periodic firing is a reproducible feature of mouse subiculum.

## Conclusion

In this study, we present evidence of spatially periodic firing in the SUB. However, their response to complex spatial manipulation or their relationship with BVC, GC, and CC needs to be determined. The SUB plays a critical role in integrating spatial codes, episodic memory, and processing dimensions related to the ongoing tasks. Further research should investigate its contributions to memory consolidation, spatial coding, and other cognitive processes.

### Significance statement

Spatial information and memory arise from interactions among hippocampal and entorhinal circuits comprising diverse, spatially tuned neurons. In this study, we provide the first evidence in mice that subicular pyramidal neurons exhibit grid-like, spatially periodic firing, a phenomenon replicated using both tetrodes and high-density silicon probes. These findings suggest that the subiculum contributes to computations beyond simple relay/integration of CA1 inputs, introducing a periodic component to subicular spatial coding that may shape downstream cortico-subcortical circuits.

## Data Availability

The raw data supporting the conclusions of this article will be made available by the authors, without undue reservation.

## Footnotes

^1^ https://github.com/cortex-lab/KiloSort

^2^ https://github.com/brendonw1/KilosortWrapper

^3^ https://github.com/kwikteam/phy

^4^ https://github.com/petersenpeter/phy2-plugins

^5^ https://cellexplorer.org

^6^ https://github.com/valegarman/HippoCookBook

^7^ https://fmatoolbox.sourceforge.net/
